# Psychiatric Symptoms and Impulsivity in Patients with Substance Use Disorders: Associations with an Aggregate ASI Interviewer Severity Score

**DOI:** 10.3390/medicina62081504

**Published:** 2026-08-05

**Authors:** Seham Mahmoud Eldeeb, Ammar Elsayed Shahtou, Maha Nabil Abobaker, Magdy Abdelhamid Elsaber, Hesham Mohamed Alrefaey, Ahmed Fathy Fadl, Eman Ahmed Alblowi, Mohamad Hussain Habil

**Affiliations:** 1 Public Health and Community Medicine Department, Zagazig University, Zagazig 44519, Egypt; 2 Population Health Management Department, Najran Health Cluster, Najran 66255, Saudi Arabia; 3 Eradah Complex Psychiatry & Addiction, Najran Health Cluster, Najran 66255, Saudi Arabia; ashahtou@moh.gov.sa (A.E.S.); mnabubakr@moh.gov.sa (M.N.A.); mahafez@moh.gov.sa (M.A.E.); helrefaey@moh.gov.sa (H.M.A.); ahmedff@moh.gov.sa (A.F.F.); 4 Strategic Planning Department, Health Holding Company, Riyadh 13521, Saudi Arabia; eman.alblowi@health.sa; 5 University Malaya Centre for Addiction Sciences, Mahsa University, Kuala Lumpur 50603, Malaysia; mohamadhussainhabil@gmail.com

**Keywords:** substance use disorders, multidomain clinical burden, psychiatric symptoms, impulsivity, Addiction Severity Index, Brief Psychiatric Rating Scale, Barratt Impulsiveness Scale-11, Saudi Arabia

## Abstract

*Background and Objectives*: Psychiatric symptoms and multidimensional impulsivity may be associated with the clinical and functional heterogeneity of substance use disorders (SUDs), but evidence from Saudi Arabian treatment populations remains limited. This study examined concurrent associations with a study-specific aggregate of Addiction Severity Index (ASI-5) interviewer severity ratings. *Materials and Methods*: This cross-sectional study included 204 adults receiving inpatient detoxification or residential rehabilitation services in Najran, Saudi Arabia. The seven ASI interviewer ratings were summed as an aggregate score. Psychiatric symptoms were assessed using the Brief Psychiatric Rating Scale (BPRS), and impulsiveness using the Barratt Impulsiveness Scale-11 (BIS-11). Analyses included age- and sex-adjusted analyses of covariance, false-discovery-rate correction, bootstrap Spearman correlations, an a priori multivariable model with HC3 robust confidence intervals, and sensitivity analyses addressing content overlap, multicollinearity, influential observations, functional form, and the bounded outcome distribution. *Results*: The mean age was 34.4 ± 7.3 years; 78.9% were male. The aggregate score was 26.33 ± 4.15. It correlated strongly with BPRS total (ρ = 0.874, 95% CI 0.827–0.908) and moderately with BIS-11 total (ρ = 0.590, 95% CI 0.494–0.669). In the primary model (adjusted R^2^ = 0.864), age, BPRS total, motor impulsiveness, and non-planning impulsiveness were conditional correlates of greater aggregate burden; male sex was inversely associated, and polysubstance use was not. BPRS remained associated after exclusion of the ASI psychiatric domain. Separate impulsivity-domain and BIS-11 total models, 5000-resample bootstrap analysis, median regression, and fractional-logit analysis retained the principal psychiatric and impulsivity directions. The polysubstance coefficient varied across sensitivity models. *Conclusions*: Psychiatric symptom burden and selected impulsivity dimensions were concurrently associated with higher study-specific aggregate ASI interviewer severity scores. Integrated psychiatric and impulsivity assessment may help characterize patients with greater concurrent multidomain clinical burden; the aggregate score should not be interpreted as a validated global severity or prediction measure.

## 1. Introduction

Substance use disorders (SUDs) are heterogeneous conditions associated with premature mortality, disability, psychiatric morbidity, family disruption, legal and occupational problems, and substantial demand on health and social services. The World Health Organization has documented a large global burden and persistent treatment gaps for alcohol and drug use disorders [1]. In Saudi Arabia, the evidence base has historically been limited and methodologically heterogeneous [2], although the Saudi National Mental Health Survey has recently provided population-based estimates and demonstrated substantial comorbidity and role impairment among people with SUD [3]. Treatment-seeking samples may differ from community populations in symptom burden, access, social consequences, and sex distribution; these contextual factors are therefore essential when interpreting clinical studies.

Psychiatric symptoms are central to the complexity of SUD. Mood, anxiety, psychotic, and behavioral symptoms may precede substance use, represent independent comorbid disorders, arise as substance-induced syndromes, or develop through chronic exposure, withdrawal, sleep disturbance, and psychosocial adversity. These pathways can be reciprocal. Depressive disorders are particularly relevant in dual-disorder practice, and personality profiles may differ between patients with SUD alone and those with comorbid major depressive disorder [4]. Symptom scales can characterize current psychiatric burden, but they do not replace structured diagnostic assessment capable of distinguishing independent disorders from substance-induced conditions [5].

Impulsivity provides a complementary psychobiological framework. It is not a unitary construct; attentional instability, acting without inhibition, limited future planning, sensation seeking, reward sensitivity, and affect-related urgency may have different mechanisms and clinical correlates [6,7,8,9]. The Barratt Impulsiveness Scale-11 (BIS-11) remains widely used and distinguishes attentional, motor, and non-planning impulsiveness [10,11]. Broader models, including Zuckerman’s Alternative Five-Factor Model and the Zuckerman–Kuhlman Personality Questionnaire, place impulsive sensation seeking within a wider personality profile that also includes neuroticism-anxiety, aggression-hostility, activity, and sociability [4]. A recent scoping review found marked heterogeneity in the measurement of impulsivity among people who use illicit drugs and supported domain-level interpretation rather than reliance on a single global construct [9]. The correspondence between self-report and behavioral impulsivity measures is also limited, and the factor structure of the BIS-11 remains debated [12,13,14]. Previous studies have linked impulsivity with addiction severity [15], treatment non-completion [16], relapse in alcohol- and heroin-dependent samples [17,18], and age at first methamphetamine use [19]. Polydrug-use patterns have also been associated with relapse [20], while depression, anxiety, impulsivity, and aggression may vary across types of drug users [21].

Sex is another important dimension. Men and women may differ in substance exposure, psychiatric comorbidity, impulsivity profiles, treatment access, and social consequences, while male-dominant treatment samples can obscure or exaggerate these differences [22]. Analyses that account for both age and sex are therefore preferable to unadjusted group comparisons.

The Addiction Severity Index (ASI) was designed as a multidimensional clinical profile covering medical, employment/support, alcohol use, drug use, legal, family/social, and psychiatric domains [23,24]. Interviewer severity ratings are clinically oriented judgments of current problem severity and treatment need within each domain, whereas domain composite scores were developed for different purposes [25,26,27]. During proposal development, the research team pre-specified an aggregate of the seven interviewer severity ratings to provide a pragmatic summary of cumulative multidomain clinical burden and support integrated clinical interpretation. Because the domains are conceptually distinct, this aggregate should be interpreted as a study-specific clinical summary rather than as a standard ASI composite, a validated unidimensional global score, or a direct measure of DSM-5 diagnostic severity [28]. Recent validation of the Arabic ASI-5 supports domain assessment in Arabic-speaking populations but does not establish the seven-domain sum as a standard ASI measure [29].

This study therefore examined concurrent associations among psychiatric symptoms, multidimensional impulsiveness, and a prespecified aggregate ASI-5 interviewer severity score among adults receiving SUD treatment in Najran, Saudi Arabia.

## 2. Materials and Methods

### 2.1. Study Design and Setting

This cross-sectional study was conducted from 1 February to 30 April 2026 at Eradah Complex for Mental Health and Addiction, Najran Health Cluster, Saudi Arabia. Participants were recruited from inpatient detoxification and residential rehabilitation services. The study was reported in accordance with the Strengthening the Reporting of Observational Studies in Epidemiology (STROBE) statement [30].

### 2.2. Participants, Diagnostic Ascertainment, and Recruitment

The target population comprised adults aged 18 years or older who had already received a clinical SUD diagnosis and had been admitted to the participating services before study recruitment. The research team did not conduct a new or independent diagnostic assessment. The established SUD diagnosis, primary substance, and polysubstance-use status were obtained from coded fields in the hospital electronic medical record (EMR), where they had been documented as part of routine clinical care by the treating psychiatric team. The principal investigator audited the extracted information against the available EMR documentation for completeness and coding consistency. The primary substance was the principal substance category documented for the index admission. Polysubstance use was coded as present when the EMR indicated use of more than one substance category; a more detailed concurrency window or substance count was not retained in the research dataset.

Eligibility criteria were age at least 18 years, an established SUD diagnosis documented in the hospital EMR, a positive urine drug-screen result in the clinical record, capacity to provide written informed consent, and ability to complete the assessments. Exclusion criteria were acute intoxication, severe withdrawal at assessment, severe cognitive impairment that interfered with participation, an active primary psychotic disorder, or inability to complete the study instruments. Other psychiatric disorders were not systematically excluded.

**Figure 1:**
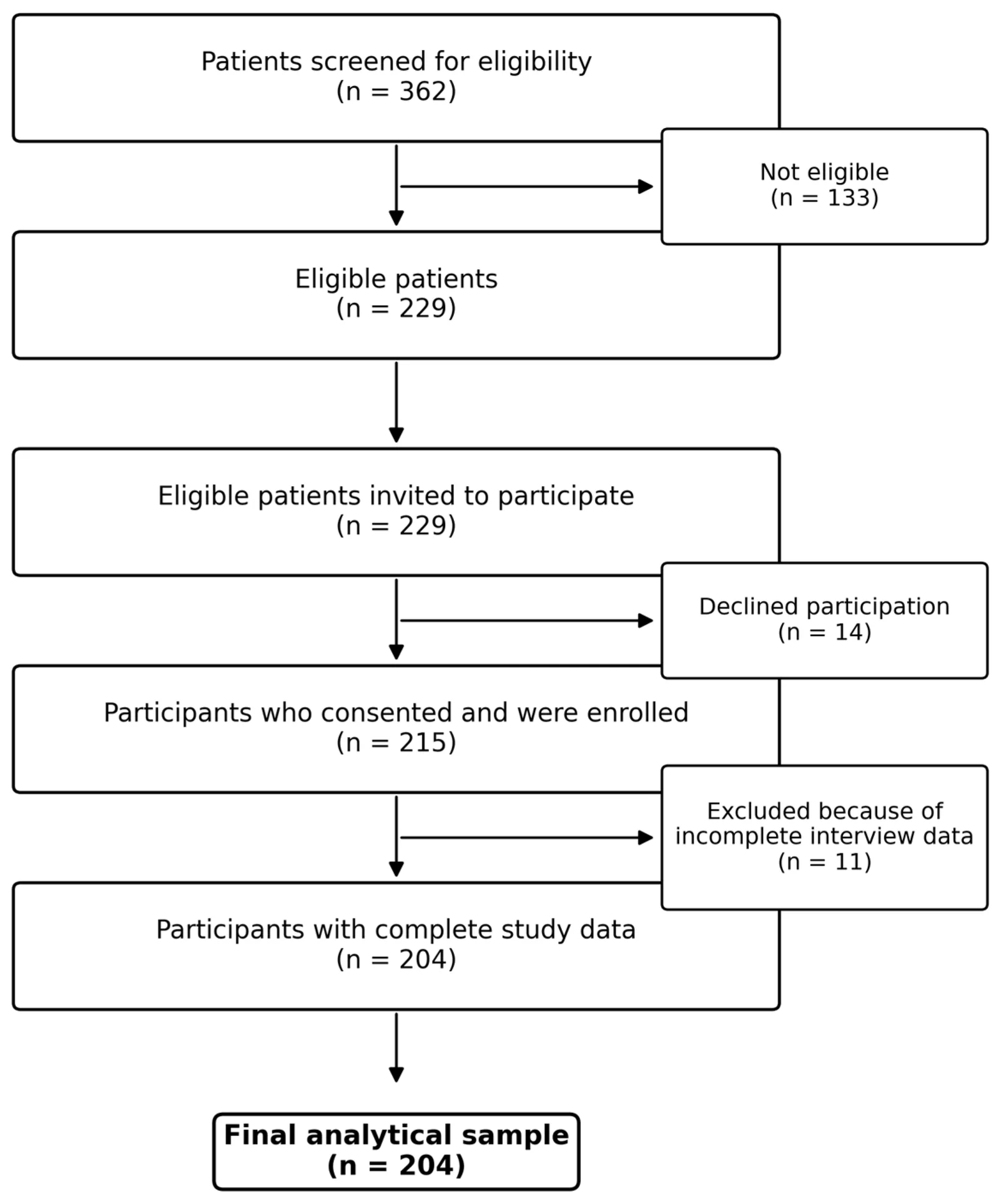
Flow of participants through screening, enrollment, and analysis.

A non-probability convenience sampling approach was used. During the study period, 362 patients were screened, 229 were eligible and invited, and 14 declined participation. Of 215 enrolled participants, 11 were excluded because of incomplete interviews. The final analytical sample comprised 204 participants (Figure 1).

### 2.3. Sample Size

No formal a priori sample-size calculation was retained. A pragmatic target of approximately 200 participants was established based on recruitment feasibility and the planned multivariable analysis. A post-collection sensitivity analysis conducted in G*Power 3.1 indicated that 204 participants provided 80% power at a two-sided alpha of 0.05 to detect a small-to-moderate multiple-regression effect (approximately Cohen’s f^2^ = 0.08) with nine candidate variables.

### 2.4. Data Collection and Quality Assurance

Data sources included structured participant assessments and the hospital EMR. Sociodemographic variables included age, recorded sex, marital status, education, employment status, and family history of SUD. Clinical variables included the EMR-documented primary substance and polysubstance-use status, together with SUD duration category and age-at-onset category. The principal investigator audited the EMR-derived variables and the linked study database for completeness, consistency, and coding accuracy. Study assessments were completed after eligibility confirmation and written informed consent.

The study instruments were administered or supervised by psychiatrists who were members of the author team. To standardize assessment, each instrument was assigned to one designated psychiatrist, who handled that instrument for all participants throughout the study. The ASI-5 and BPRS were interviewer-administered by their respective designated psychiatrists. The BIS-11 was completed by participants as a self-report measure under standardized supervision by its designated psychiatrist, who checked completeness. Because each instrument had a single designated psychiatrist across the entire sample, inter-rater reliability was not applicable.

Completed assessments were entered directly into a structured Google Form, with the medical record number used as a restricted unique entry identifier. The principal investigator monitored the linked database and reviewed entries for completeness, consistency, and entry accuracy. Before reanalysis, the locked dataset was audited against scale-scoring rules and table totals.

### 2.5. Measures

Addiction-related problems were assessed using the Arabic ASI-5 [23,24,29]. The ASI covers seven clinically distinct domains: medical, employment/support, alcohol use, drug use, legal, family/social, and psychiatric status. Interviewer severity ratings range from 0 to 9, and all seven domains were reported separately. The prespecified primary outcome was an aggregate ASI interviewer severity score, calculated by summing the seven interviewer ratings (possible range 0–63). This arithmetic sum was selected during study-proposal development as a pragmatic description of cumulative multidomain problem burden. It has no established factor structure, psychometric validation, or accepted weighting scheme as a global severity construct. It is not a standard ASI composite, a validated unidimensional global measure, or equivalent to DSM-5 SUD severity [25,26,27].

Current psychiatric symptoms were assessed with the 18-item Brief Psychiatric Rating Scale (BPRS) [31]. Total and positive, negative, affective, activation, and resistance domain scores were calculated according to Shafer’s factor structure [32]. The BPRS quantified symptom burden at the time of assessment; it was not used to diagnose dual disorders or to distinguish independent psychiatric disorders from substance-induced symptoms.

Impulsiveness was assessed with the 30-item BIS-11 [10,11]. The total score and attentional, motor, and non-planning second-order domain scores were calculated using standard scoring procedures. Higher scores indicate greater self-reported impulsiveness. Arabic psychometric evidence has raised concerns about the factor structure of the full BIS-11 and supports explicit reporting of sample-specific reliability and domain-level results [33].

The locked analytical file contained the seven ASI interviewer ratings and BPRS and BIS-11 domain totals, but not the individual BPRS or BIS-11 item responses. Cronbach’s alpha is not meaningful for a single ASI domain rating, and item-level BPRS and BIS-11 internal-consistency coefficients could not be independently reproduced from the locked file. Accordingly, no alpha coefficients are reported in this revision.

### 2.6. Statistical Analysis

Completed assessments were entered directly into Google Forms (Google LLC, Mountain View, CA, USA). The numerical audit and analyses were performed using Python 3.13 (Python Software Foundation, Wilmington, DE, USA) with pandas 2.2.3, SciPy 1.17.0, and statsmodels 0.14.6. The reproducible analysis script, including the fixed random seed (20260729), is provided . Continuous variables were summarized with means, standard deviations, and observed ranges, and categorical variables with frequencies and percentages. All analyses used complete cases. A post-collection sensitivity analysis was conducted using G*Power version 3.1 (Heinrich Heine University Düsseldorf, Düsseldorf, Germany).

Age- and sex-adjusted associations were examined using analysis of covariance. Age was entered continuously and sex as a fixed factor. For each additional characteristic, the model included age, sex, and the characteristic under examination. The sex model included age as the covariate; the age model included sex as the factor. Effect sizes were reported using partial η^2^. Because the table contained multiple exploratory comparisons, Benjamini–Hochberg false-discovery-rate correction was applied separately within each outcome [34,35]. Duration of SUD and age at onset were available only as the original categories (<10 versus at least 10 years and <20 versus at least 20 years); unsupported continuous means from earlier drafts were removed.

Spearman rank correlations were used to assess associations of BPRS and BIS-11 total and domain scores with the aggregate problem-burden score. Bootstrap 95% confidence intervals and false-discovery-rate-adjusted *p* values were reported. The primary multivariable linear model was specified on clinical grounds rather than selected by bivariate *p* values [36]. It included age, sex, polysubstance use, BPRS total, BIS-11 motor, and BIS-11 non-planning scores. Age was retained instead of simultaneously entering age, duration, and age at onset because these variables are structurally related and the available duration/onset variables were dichotomized. Unstandardized and standardized coefficients, HC3 heteroskedasticity-consistent standard errors and confidence intervals, *p* values, variance inflation factors, the intercept, model F statistic, R^2^, adjusted R^2^, residual standard error, and analytical n were reported [37].

Model diagnostics included residual-versus-fitted and normal Q-Q plots, the Shapiro–Wilk and Breusch–Pagan tests, leverage, Cook’s distance, variance inflation factors, and the Ramsey RESET test. Post hoc sensitivity analyses requested during peer review addressed multicollinearity and coefficient stability by fitting separate models containing BIS-11 motor or non-planning impulsiveness, a model replacing both domains with BIS-11 total, sequential covariate blocks, and 5000 nonparametric bootstrap resamples of the primary coefficients.

Additional robustness analyses included median regression; a fractional-logit quasi-likelihood model applied to the aggregate score divided by 63, with a logit link and HC3 robust confidence intervals [38]; exclusion of observations with Cook’s distance greater than 4/n; exclusion of one record from each identical pair; and a quadratic-term model for age, BPRS total, BIS-11 motor, and BIS-11 non-planning scores. Predictor-specific leave-one-domain-out analyses removed ASI psychiatric status for BPRS, alcohol and drug domains for polysubstance use, and employment/support for employment. Because the design was cross-sectional, all coefficients were interpreted as conditional concurrent associations rather than causal effects, prognostic estimates, or measures of predictive validity [39].

### 2.7. Ethical Considerations

This study was conducted in accordance with the Declaration of Helsinki [40] and approved by the Institutional Review Board of Najran Health Cluster (approval number 2026-90A; 1 February 2026). Written informed consent was obtained before enrollment. Participation was voluntary; refusal or withdrawal did not affect access to care; access to the database was restricted to authorized research-team members; and identifying information was excluded from study reports.

## 3. Results

### 3.1. Participant Flow and Characteristics

Among 362 patients screened for eligibility, 133 were ineligible, and 229 were eligible and invited to participate. Fourteen declined participation. Of 215 enrolled participants, 11 were excluded because of incomplete interview data, resulting in a final analytical sample of 204 participants (Figure 1). No values were missing among the participants analyzed.

The mean age was 34.4 ± 7.3 years. Most participants were male (78.9%), single (70.6%), educated to the secondary level (73.0%), and unemployed (75.0%). Cannabis was the most frequently recorded primary substance (34.8%), followed by alcohol (19.6%), tramadol (17.2%), pregabalin (16.7%), and stimulants (11.8%). Polysubstance use was recorded in 38.2%; 45.6% had an SUD duration of at least 10 years, and 48.5% had onset before 20 years of age (Table 1).

### 3.2. Scale Scores

The study-specific aggregate problem-burden score had a mean of 26.33 ± 4.15 (range 15–41). The psychiatric-status domain had the highest mean rating (6.55 ± 1.52), followed by alcohol use (4.47 ± 2.47), family/social relationships (4.40 ± 1.61), and employment/support (4.37 ± 1.79). The mean BPRS total was 42.04 ± 8.45, and the mean BIS-11 total was 63.28 ± 13.57 (Table 2). Table 2 reports score distributions only; alpha coefficients were removed for the psychometric reasons stated in the Methods section.

### 3.3. Age- and Sex-Adjusted Analyses

After adjustment for sex, age was positively associated with the aggregate problem-burden score and BPRS total but negatively associated with BIS-11 total (all false-discovery-rate-adjusted *p* < 0.01). After adjustment for age, sex was not associated with aggregate burden or BPRS total; however, men had a higher adjusted BIS-11 mean than women (66.55 versus 51.06; adjusted *p* < 0.001) (Table 3).

Employment status, primary substance, and polysubstance use were strongly associated with the outcomes in age- and sex-adjusted analyses. Yet the leave-one-domain-out and model-stability results demonstrated why the aggregate outcome requires caution. The unemployment association disappeared after removal of the ASI employment/support domain, indicating substantial shared content. The polysubstance coefficient was nonsignificant in the primary, motor-only, BIS-total, bootstrap, and fractional-logit analyses, but positive in the non-planning-only, median-regression, influence-exclusion, and alcohol/drug-domain-exclusion models. This variability precludes a simple conclusion that polysubstance use either does or does not have an independent association with a global severity construct. Primary-substance differences were exploratory and may reflect treatment selection, routine clinical classification, and toxicology eligibility rather than substance-specific effects.

### 3.4. Correlation Analysis

The ASI Aggregate burden score correlated strongly with BPRS total (Spearman ρ = 0.874, bootstrap 95% CI 0.827–0.908) and moderately with BIS-11 total (ρ = 0.590, 95% CI 0.494–0.669). Among BPRS dimensions, the strongest correlation was observed for positive symptoms (ρ = 0.757), followed by affective symptoms (ρ = 0.662) and negative symptoms (ρ = 0.592). Among BIS-11 dimensions, attentional impulsiveness showed the strongest bivariate correlation (ρ = 0.721), followed by motor (ρ = 0.564) and non-planning impulsiveness (ρ = 0.469). All correlations remained significant after false-discovery-rate correction (Table 4).

### 3.5. Multivariable Analysis and Diagnostics

The primary model explained 86.8% of the variance in the ASI aggregate problem-burden score (adjusted R^2^ = 0.864; F[6,197] = 216.00; *p* < 0.001). Within the specified model, higher age, BPRS total, BIS-11 motor impulsiveness, and BIS-11 non-planning impulsiveness were positive conditional correlates of higher burden. Male sex was inversely associated. Polysubstance use was not conditionally associated after adjustment for psychiatric symptoms and impulsivity measures. The largest standardized coefficient was observed for BPRS total (beta = 0.495), followed by age (beta = 0.329), motor impulsiveness (beta = 0.327), male sex (beta = −0.263), and non-planning impulsiveness (beta = 0.198) (Table 5).

Residuals departed from normality (Shapiro–Wilk *p* < 0.001), but the Breusch–Pagan test did not indicate heteroskedasticity (*p* = 0.195). HC3 robust confidence intervals were therefore reported. Variance inflation factors ranged from 1.79 to 6.75, with the highest value for BIS-11 motor impulsiveness, consistent with the strong correlation between motor and non-planning scores. Nine observations exceeded the conventional Cook’s-distance threshold of 4/n, but none had Cook’s distance above 1; the maximum was 0.106. Diagnostic plots and the predictor correlation matrix are provided in the Supplementary Materials.

### 3.6. Sensitivity Analyses

After exclusion of the ASI psychiatric-status domain, BPRS total remained conditionally associated with the modified six-domain outcome (B = 0.167, 95% CI 0.125–0.209; *p* < 0.001). After exclusion of the ASI alcohol- and drug-use domains, polysubstance use was associated with the modified outcome (B = 3.275, 95% CI 2.519–4.031; *p* < 0.001). In contrast, the employment coefficient was attenuated and became nonsignificant after removal of the employment/support domain (B = −0.579, 95% CI −1.484 to 0.325; *p* = 0.210). These changes are consistent with predictor-outcome content overlap (Supplementary Table S2).

The motor-only model retained a positive motor coefficient (B = 0.336, 95% CI 0.262–0.410; *p* < 0.001), the non-planning-only model retained a positive non-planning coefficient (B = 0.262, 95% CI 0.203–0.322; *p* < 0.001), and the model using BIS-11 total produced a positive total-score coefficient (B = 0.165, 95% CI 0.135–0.196; *p* < 0.001). The inverse male and positive BPRS coefficients persisted in all three models, while maximum VIF decreased to 4.04 or lower. In sequential models, the polysubstance coefficient attenuated from 7.145 in the demographic/clinical model to 2.657 after adding BPRS and to 0.534 after adding the BIS-11 domains. In 5000 bootstrap resamples, the 95% intervals for age, male sex, BPRS, motor, and non-planning coefficients excluded zero, whereas the polysubstance interval crossed zero (−0.244 to 1.244) (Supplementary Tables S3–S5).

Median regression and fractional-logit analyses retained the positive age, BPRS, motor, and non-planning directions and the inverse male coefficient. Polysubstance use was positive in median regression (B = 1.101, 95% CI 0.138–2.063; *p* = 0.025) but nonsignificant in fractional logit (*p* = 0.213). After exclusion of nine observations with Cook’s distance greater than 4/n, the principal psychiatric and impulsivity coefficients remained positive, while polysubstance use became positive (B = 0.831, 95% CI 0.165–1.496; *p* = 0.014). The RESET test did not indicate general misspecification (*p* = 0.856), but the joint quadratic-term test was significant (*p* = 0.019), driven mainly by curvature in the BIS-11 motor association (quadratic *p* = 0.003). The linear motor coefficient should therefore be interpreted as an average association across the observed range, and the polysubstance coefficient as model-sensitive (Supplementary Tables S6 and S7).

## 4. Discussion

In this cross-sectional study of 204 treatment-seeking adults with SUD, current psychiatric symptom burden and selected impulsivity dimensions were strongly associated with a prespecified aggregate ASI interviewer severity score. BPRS total had the largest standardized coefficient in the primary model, followed by age and motor impulsiveness. The psychiatric and impulsivity directions were retained across separate-domain, BIS-11 total, bootstrap, median-regression, fractional-logit, duplicate-exclusion, and influence-exclusion analyses. However, the polysubstance coefficient varied across model specifications, and quadratic analysis suggested that the motor association was not completely linear. These findings support cautious, model-aware interpretation rather than ranking the coefficients as separable causal effects.

The strong association between BPRS total and aggregate problem burden is consistent with evidence that psychiatric symptoms accompany greater clinical and functional difficulty among people receiving SUD treatment [41,42]. Positive, affective, and negative symptom dimensions showed substantial bivariate associations. However, the BPRS captures current symptoms rather than formal psychiatric diagnoses. Symptoms may reflect independent mood, anxiety, or psychotic disorders, substance-induced states, withdrawal, sleep disruption, or treatment context. The study by Marquez-Arrico and colleagues illustrates how comorbid depression and wider personality characteristics can distinguish clinically meaningful subgroups within SUD treatment populations [4]. The persistence of the BPRS association after removal of the ASI psychiatric domain reduces, but does not eliminate, concerns about common-method and common-rater covariance.

The impulsivity findings support multidimensional assessment. Motor and non-planning impulsiveness were positive conditional correlates in the primary model, and each remained associated when entered separately; BIS-11 total was also associated in an alternative model. Nevertheless, motor and non-planning scores were strongly correlated, and the quadratic sensitivity analysis suggested curvature in the motor relationship. Their simultaneous linear coefficients therefore should not be interpreted as fully distinct mechanistic effects. Recent clinical work likewise depicts impulsivity as a system of partially overlapping self-report and neurocognitive dimensions, while treatment reviews emphasize domain-specific rather than global interpretation [43,44]. The 2025 scoping review by Makarenko and colleagues similarly showed that observed associations depend on the impulsivity construct and instrument [9]. Debates about BIS-11 structure and the modest convergence of self-report with behavioral tasks further limit claims about inhibitory control [12,13,14,33].

Age and sex showed complex relationships. Older age was associated with greater aggregate and psychiatric burden but lower BIS-11 total in adjusted analyses. Sex was not associated with aggregate burden or BPRS total after age adjustment, although men had higher BIS-11 scores. In the multivariable models, male sex was inversely associated with aggregate burden, and this direction persisted across the principal sensitivity analyses and bootstrap resampling. However, its magnitude changed across sequential models, and the sample included only 43 women. The coefficient is therefore best viewed as a conditional sample-specific association affected by correlated covariates, not as evidence that women generally experience greater addiction-related burden. The broader literature supports examining sex and gender as potential modifiers rather than assuming uniform effects [22].

Employment status, primary substance, and polysubstance use were strongly associated with the outcomes in age- and sex-adjusted analyses. Yet the leave-one-domain-out results demonstrated why the aggregate outcome requires caution. The unemployment association disappeared after removal of the ASI employment/support domain, indicating that shared content largely generated the original association. Polysubstance use was nonsignificant in the primary multivariable model but became significant when the alcohol- and drug-use domains were removed. This unexpected sensitivity suggests that relationships between substance patterns and the equally weighted aggregate cannot be summarized as a simple increase in global severity. Primary-substance differences were global exploration associations and may also reflect treatment selection, clinical classification, and toxicology eligibility rather than substance-specific causal effects.

Clinically, the results support integrated assessment rather than use of the aggregate score as a risk instrument. Current psychiatric symptoms and domain-level impulsivity may help characterize concurrent needs related to engagement, behavioral regulation, planning, and coordination between addiction and mental-health services. Recent peer-reviewed synthesis and the current ASAM addiction-medicine text similarly emphasize integrated assessment of co-occurring psychiatric conditions, substance-specific diagnosis, and individualized treatment planning [45,46]. The measures should complement, not replace, structured psychiatric diagnosis, DSM-5 substance-specific severity assessment, evaluation of intoxication and withdrawal, medication review, and direct assessment of social and functional needs. The present study did not measure prospective relapse, retention, adherence, or recovery; therefore, risk stratification and treatment-outcome claims would be inappropriate [39,47].

The primary outcome requires particular caution. It is an equal-weighted arithmetic sum of seven single interviewer ratings designed to represent distinct clinical domains and treatment needs. No factor analysis, external validation, or accepted ASI scoring convention supports treating this sum as a unidimensional latent severity construct. BPRS, employment, and substance-pattern variables also overlap conceptually with the psychiatric, employment/support, and alcohol/drug components of the outcome. Leave-one-domain-out analyses reduce direct overlap but cannot eliminate common-method, common-rater, and construct contamination. Accordingly, the score should be interpreted only as a study-specific indicator of concurrent multidomain problem burden in this sample, not as a psychometrically established global addiction-severity measure [25,26,27].

The current study has several strengths. It contributes data from an underrepresented Saudi treatment population, includes recognized multidimensional clinical instruments, examines impulsivity at the domain level, and uses an audited dataset with age- and sex-adjusted analyses, false-discovery-rate correction, robust confidence intervals, complete primary-model reporting, content-overlap analyses, separate and total-score impulsivity models, sequential models, bootstrap coefficient-stability analysis, and alternative outcome models. Administration was standardized by assigning one designated psychiatrist to each interviewer-administered instrument for the entire sample, and EMR-derived diagnosis and substance-use classifications were audited by the principal investigator.

The limitations are substantial. The primary outcome is unvalidated, equally weighted, and content-overlapping, and no reported sensitivity analysis can convert it into a standard ASI composite or validated global measure. Item-level BPRS and BIS-11 responses were not retained in the locked analytical file, so item-level internal consistency could not be independently reproduced. The cross-sectional design precludes temporal, causal, prognostic, and predictive inference [39]. Convenience recruitment from a single treatment complex, recent toxicology positivity, and the predominantly male sample limit transportability to community, outpatient, untreated, and female populations. SUD diagnosis and substance-use classifications came from routine-care EMR documentation and were not independently re-established with a structured diagnostic interview; independent psychiatric comorbidity could not be systematically distinguished from substance-induced syndromes, and active primary psychotic disorder was excluded. Duration and age at onset were retained only as categories. Residuals were non-normal, VIF reached 6.75, nine observations exceeded 4/n for Cook’s distance, and the motor association showed evidence of nonlinearity. Although HC3, median regression, fractional logit, bootstrap, and influence analyses supported the principal psychiatric and impulsivity directions, the polysubstance coefficient was model-sensitive. Finally, two exact data-profile pairs lacked identifiers that could establish whether they represented duplicate entries or distinct participants; retaining or removing one record from each pair did not materially change the primary psychiatric and impulsivity estimates.

## 5. Conclusions

Among adults receiving SUD treatment in Najran, greater current psychiatric symptom burden, motor impulsiveness, non-planning impulsiveness, and older age were concurrent conditional correlates of a higher study-specific aggregate ASI interviewer severity score. The psychiatric and impulsivity directions were retained across multiple sensitivity analyses, although the motor relationship showed curvature and the polysubstance coefficient was model-sensitive. Content-overlap analyses also showed that some apparent relationships, particularly employment, depended on the domains included in the aggregate. The score should therefore be interpreted as an unvalidated, sample-specific summary of concurrent multidomain problem burden and not as a standard ASI composite, global addiction-severity measure, or prediction tool. Integrated psychiatric, behavioral, and functional assessment may help characterize patients with greater concurrent burden, while future multisite longitudinal studies should use substance-specific DSM-5 severity, validated ASI domain outcomes, structured dual-disorder diagnosis, balanced sex representation, and repeated clinical outcomes.

## Figures and Tables

**Table 1:** Sociodemographic and clinical characteristics of the analytical sample (N = 204).

Variable	Category/Summary	Value
Age, years	Mean ± SD	34.4 ± 7.3
Sex	Male	161 (78.9)
	Female	43 (21.1)
Marital status	Single	144 (70.6)
	Married	60 (29.4)
Education	Secondary education	149 (73.0)
	Higher education	55 (27.0)
Employment	Employed	51 (25.0)
	Unemployed	153 (75.0)
Family history of SUD	Positive	32 (15.7)
	Negative	172 (84.3)
Primary substance	Cannabis	71 (34.8)
	Alcohol	40 (19.6)
	Tramadol	35 (17.2)
	Pregabalin	34 (16.7)
	Stimulants	24 (11.8)
Duration of SUD	<10 years	111 (54.4)
	≥10 years	93 (45.6)
Age at onset	<20 years	99 (48.5)
	≥20 years	105 (51.5)
Polysubstance use	Yes	78 (38.2)
	No	126 (61.8)

**Table 2:** ASI interviewer ratings, BPRS scores, and BIS-11 scores.

Scale	Domain/Score	Mean ± SD	Range
ASI	Medical	1.45 ± 1.22	0–4
	Employment/support	4.37 ± 1.79	1–7
	Alcohol-use	4.47 ± 2.47	0–8
	Drug-use	3.07 ± 1.93	0–6
	Legal	2.01 ± 0.65	1–4
	Family/social	4.40 ± 1.61	2–8
	Psychiatric	6.55 ± 1.52	4–9
	Aggregate Score	26.33 ± 4.15	15–41
BPRS	Positive symptoms	12.61 ± 4.61	5–20
	Negative symptoms	6.33 ± 1.18	4–8
	Affective symptoms	11.93 ± 3.72	5–18
	Activation	5.13 ± 0.89	3–7
	Resistance	6.03 ± 1.20	3–9
	Total	42.04 ± 8.45	26–57
BIS-11	Attentional	16.96 ± 2.72	13–24
	Motor	21.87 ± 6.10	13–33
	Non-planning	24.46 ± 5.80	16–35
	Total	63.28 ± 13.57	43–91

**Table 3:** Age- and sex-adjusted analyses of covariance.

Characteristic	ASI Aggregate Burden			BPRS			BIS-11		
	F	FDR *p*	Partial η^2^	F	FDR *p*	Partial η^2^	F	FDR *p*	Partial η^2^
Age	23.07	<0.001	0.103	11.90	0.001	0.056	16.26	<0.001	0.075
Sex	0.21	0.722	0.001	1.58	0.211	0.008	60.01	<0.001	0.230
Marital status	12.61	0.001	0.059	4.04	0.065	0.020	78.14	<0.001	0.281
Education level	1.20	0.343	0.006	19.10	<0.001	0.087	3.03	0.104	0.015
Employment status	92.74	<0.001	0.317	25.66	<0.001	0.114	89.61	<0.001	0.309
Family history of SUD	0.02	0.887	0.000	1.90	0.196	0.009	1.84	0.196	0.009
Primary substance	29.74	<0.001	0.376	101.01	<0.001	0.672	18.97	<0.001	0.278
Duration of SUD	3.02	0.120	0.015	1.84	0.196	0.009	0.16	0.688	0.001
Age at onset	20.70	<0.001	0.094	61.76	<0.001	0.236	3.05	0.104	0.015
Polysubstance use	314.81	<0.001	0.612	303.48	<0.001	0.603	248.70	<0.001	0.554

Note: Age was modeled continuously. Sex was included as a fixed factor. For other characteristics, the model included age, sex, and the characteristic under examination. FDR *p* values are Benjamini–Hochberg adjusted within each outcome.

**Table 4:** Spearman correlations with the ASI Aggregate burden score.

Measure	Spearman ρ	Bootstrap 95% CI	FDR-Adjusted *p*
BPRS positive	0.757	0.691–0.807	<0.001
BPRS negative	0.592	0.481–0.686	<0.001
BPRS affective	0.662	0.561–0.744	<0.001
BPRS activation	0.497	0.366–0.607	<0.001
BPRS resistance	0.333	0.196–0.457	<0.001
BPRS total	0.874	0.827–0.908	<0.001
BIS-11 attentional	0.721	0.651–0.781	<0.001
BIS-11 motor	0.564	0.474–0.646	<0.001
BIS-11 non-planning	0.469	0.349–0.577	<0.001
BIS-11 total	0.590	0.494–0.669	<0.001

**Table 5:** Factors independently associated with the ASI Aggregate burden score.

Variable	B	HC3 SE	Standardized Beta	HC3 95% CI	*p*	VIF
Intercept	3.275	1.387	-	0.540 to 6.010	0.019	-
Age, years	0.186	0.022	0.329	0.143 to 0.230	<0.001	1.79
Male sex	−2.673	0.356	−0.263	−3.376 to −1.970	<0.001	2.14
Polysubstance use	0.534	0.387	0.063	−0.230 to 1.298	0.169	4.09
BPRS total	0.243	0.022	0.495	0.199 to 0.287	<0.001	3.11
BIS-11 motor	0.222	0.041	0.327	0.141 to 0.303	<0.001	6.75
BIS-11 non-planning	0.142	0.028	0.198	0.087 to 0.197	<0.001	4.11

Model *n* = 204; F(6,197) = 216.00; *p* < 0.001; R^2^ = 0.868; adjusted R^2^ = 0.864; residual standard error = 1.53. Reference categories: female and no polysubstance use. B = unstandardized coefficient; HC3 = heteroskedasticity-consistent robust inference; VIF = variance inflation factor.

## Data Availability

Data is unavailable due to privacy and ethical restrictions.

## Supplementary Materials

The following supporting information can be downloaded at: https://www.mdpi.com/article/10.3390/medicina62081504/s1, Supplementary Table S1 presents the numerical audit trail; Table S2 presents leave-one-domain-out analyses; Tables S3–S5 present multicollinearity, sequential-model, and bootstrap coefficient-stability analyses; Table S6 presents median-regression, fractional-logit, influence-exclusion, and duplicate-exclusion estimates; Table S7 presents model diagnostics and functional-form checks; Table S8 presents the predictor correlation matrix; Figures S1 and S2 show the residual-versus-fitted and normal Q-Q plots. Supplementary File provides the reproducible Python analysis script.

## Abbreviations

The following abbreviations are used in this manuscript:

SUD	Substance Use Disorders
ASI	Addiction Severity Index
BPRS	Brief Psychiatric Rating Scale
BIS-11	Barratt Impulsiveness Scale-11
CI	Confidence interval
FDR	False discovery rate
HC3	Heteroskedasticity-consistent covariance estimator
VIF	Variance inflation factor
