# Supplementary material for: Psychiatric Symptoms and Impulsivity in Patients with Substance Use Disorders: Associations with an Aggregate ASI Interviewer Severity Score
Source: Medicina (Kaunas). 2026 Aug 5;62(8):1504. doi: 10.3390/medicina62081504 (PMC13515123; doi:10.3390/medicina62081504)

Supplementary Material

Psychiatric Symptoms and Impulsivity in Patients with Substance Use Disorders: Associations with an Aggregate ASI Interviewer Severity Score

Supplementary Table S1. Numerical audit trail

Component	Original manuscript	Locked-data audit	Records affected	Audit action	Outputs affected
Analysis population	N = 204	N = 204; no missing values	0	All 204 records retained in the primary analysis	All tables regenerated from one locked dataset
Age	35.5 ± 9.7 years	34.4 ± 7.3 years	0 raw age cells	Prior summary was not reproducible; mean and SD recomputed from all 204 age values	Abstract, Results, Table 1
Aggregate ASI interviewer score	26.8 ± 6.7	26.33 ± 4.15; all totals matched the seven domains	0 raw ASI cells	Prior summary was not reproducible; all descriptive and inferential results regenerated	Abstract, Tables 2-5, Discussion
BPRS total	42.0 ± 9.7	42.04 ± 8.45	1 record	Excel row 85 corrected from 18 to 41 because the five BPRS domains summed to 41	Abstract, Tables 2-5, regression conclusions
BIS-11 total	66.1 ± 12.4	63.28 ± 13.57; all totals matched the three domains	0 raw BIS-11 cells	Prior summary was not reproducible; all results regenerated	Abstract, Tables 2-5, Discussion
Duration and age at onset	Continuous means and dichotomies appeared in earlier drafts	Locked file retained only <10/≥10 years and <20/≥20 years categories	0	Unsupported continuous values removed; categories excluded from the primary regression	Methods, Table 1, regression specification
Categorical coding	Coding incompletely documented	Codebook verified: 1/2 coding for binary variables and 1-5 for primary substance	204 analytic recodes; no raw values changed	Temporary 0/1 indicators created for regression; reference categories documented	Methods, table notes, analysis script
Exact profile duplicates	Not documented	Two identical pairs: participant rows 17/21 and 77/78	4 records flagged	Retained because identifiers were unavailable; sensitivity analysis removed one record per pair (n = 202)	Supplementary Table S6
Cronbach alpha	ASI and domain-specific coefficients were reported	Single ASI ratings do not support alpha; item-level BPRS/BIS-11 responses were not retained	Not applicable	All alpha coefficients removed to avoid psychometric overinterpretation	Methods and Table 2
Regression and conclusions	Original coefficients and conclusions were not reproducible from the locked file	Primary and sensitivity models regenerated after audit	All 204 records; one corrected BPRS total	All regression tables and corresponding statements replaced	Abstract, Results, Discussion, Conclusion
Software and reproducibility	Not fully documented	Python 3.13; pandas 2.2.3; SciPy 1.17.0; statsmodels 0.14.6; seed 20260729	Not applicable	Round2_Analysis_Reproducible.py regenerates the audit, models, tables, and plots	Supplementary File S1

Note: A raw record count of zero means that the participant-level values were not altered; the manuscript summary was nevertheless replaced because it could not be reproduced from the locked participant-level file. The single participant-level correction was the BPRS total in Excel row 85 (18 to 41).

Supplementary Table S2. Leave-one-domain-out sensitivity analyses

Outcome specification	Target variable	B	HC3 95% CI	p
Primary aggregate	BPRS total	0.243	0.199 to 0.287	<0.001
Psychiatric domain excluded	BPRS total	0.167	0.125 to 0.209	<0.001
Primary aggregate	Polysubstance use	0.534	-0.225 to 1.294	0.168
Alcohol and drug domains excluded	Polysubstance use	3.275	2.519 to 4.031	<0.001
Aggregate includes employment/support	Unemployed	3.049	2.090 to 4.008	<0.001
Employment/support domain excluded	Unemployed	-0.579	-1.484 to 0.325	0.210

Note: Every model included age, sex, polysubstance use, BPRS total, BIS-11 motor, and BIS-11 non-planning unless the target predictor required adding employment. The results illustrate direct content overlap; they do not validate the aggregate outcome.

Supplementary Table S3. Sensitivity models addressing correlated BIS-11 domains

Model	n	R^2	Adjusted R^2	Maximum VIF
Primary: motor + non-planning	204	0.868	0.864	6.75
Motor only	204	0.858	0.855	4.04
Non-planning only	204	0.852	0.848	3.81
BIS-11 total	204	0.878	0.875	3.93
**Model**	**Variable**	**B**	**HC3 95% CI**	**p**
Primary: motor + non-planning	Intercept	3.275	0.556 to 5.993	0.018
Primary: motor + non-planning	Age	0.186	0.144 to 0.229	<0.001
Primary: motor + non-planning	Male sex	-2.673	-3.371 to -1.974	<0.001
Primary: motor + non-planning	Polysubstance use	0.534	-0.225 to 1.294	0.168
Primary: motor + non-planning	BPRS total	0.243	0.199 to 0.287	<0.001
Primary: motor + non-planning	BIS-11 motor	0.222	0.142 to 0.303	<0.001
Primary: motor + non-planning	BIS-11 non-planning	0.142	0.087 to 0.196	<0.001
Motor only	Intercept	5.141	2.504 to 7.777	<0.001

Model	n	R²	Adjusted R²	Maximum VIF
Motor only	Age	0.176	0.131 to 0.222	<0.001
Motor only	Male sex	-2.746	-3.460 to -2.032	<0.001
Motor only	Polysubstance use	0.719	-0.032 to 1.471	0.061
Motor only	BPRS total	0.230	0.188 to 0.272	<0.001
Motor only	BIS-11 motor	0.336	0.262 to 0.410	<0.001
Non-planning only	Intercept	4.026	1.036 to 7.015	0.008
Non-planning only	Age	0.151	0.114 to 0.188	<0.001
Non-planning only	Male sex	-1.973	-2.613 to -1.334	<0.001
Non-planning only	Polysubstance use	1.101	0.207 to 1.995	0.016
Non-planning only	BPRS total	0.282	0.237 to 0.327	<0.001
Non-planning only	BIS-11 non-planning	0.262	0.203 to 0.322	<0.001
BIS-11 total	Intercept	2.192	-0.589 to 4.974	0.122
BIS-11 total	Age	0.187	0.148 to 0.226	<0.001
BIS-11 total	Male sex	-2.761	-3.411 to -2.111	<0.001
BIS-11 total	Polysubstance use	0.594	-0.156 to 1.345	0.120
BIS-11 total	BPRS total	0.219	0.179 to 0.259	<0.001
BIS-11 total	BIS-11 total	0.165	0.135 to 0.196	<0.001

Note: The maximum VIF fell from 6.75 in the primary domain model to 4.04 or lower in the alternative impulsivity models. The positive psychiatric and impulsivity coefficients and inverse male coefficient persisted.

Supplementary Table S4. Sequential models showing coefficient changes

Model	Variable	B	HC3 95% CI	p	Adjusted R²
Model 1: age, sex, polysubstance	Age	0.070	0.024 to 0.116	0.003	0.646
Model 1: age, sex, polysubstance	Male sex	-3.723	-4.511 to -2.935	<0.001	0.646
Model 1: age, sex, polysubstance	Polysubstance use	7.145	6.332 to 7.957	<0.001	0.646

Model	Variable	B	HC3 95% CI	p	Adjusted R²
Model 2: + BPRS total	Age	0.057	0.021 to 0.094	0.002	0.790
Model 2: + BPRS total	Male sex	-1.039	-1.691 to -0.388	0.002	0.790
Model 2: + BPRS total	Polysubstance use	2.657	1.803 to 3.510	<0.001	0.790
Model 2: + BPRS total	BPRS total	0.304	0.252 to 0.355	<0.001	0.790
Model 3: + BIS domains	Age	0.186	0.144 to 0.229	<0.001	0.864
Model 3: + BIS domains	Male sex	-2.673	-3.371 to -1.974	<0.001	0.864
Model 3: + BIS domains	Polysubstance use	0.534	-0.225 to 1.294	0.168	0.864
Model 3: + BIS domains	BPRS total	0.243	0.199 to 0.287	<0.001	0.864
Model 3: + BIS domains	BIS-11 motor	0.222	0.142 to 0.303	<0.001	0.864
Model 3: + BIS domains	BIS-11 non-planning	0.142	0.087 to 0.196	<0.001	0.864

Note: Model 1 included age, sex, and polysubstance use; Model 2 added BPRS total; Model 3 added BIS-11 motor and non-planning. The polysubstance coefficient attenuated from 7.145 to 2.657 to 0.534.

Supplementary Table S5. Nonparametric bootstrap coefficient-stability analysis

Variable	Primary B	Bootstrap 95% percentile interval	Bootstrap median	Same-sign resamples
Intercept	3.275	0.429 to 5.837	3.276	98.6%
Age	0.186	0.146 to 0.230	0.187	100.0%
Male sex	-2.673	-3.344 to -1.967	-2.660	100.0%
Polysubstance use	0.534	-0.244 to 1.244	0.524	91.3%
BPRS total	0.243	0.201 to 0.288	0.243	100.0%
BIS-11 motor	0.222	0.141 to 0.303	0.223	100.0%
BIS-11 non-planning	0.142	0.086 to 0.196	0.141	100.0%

Note: Five thousand participant-level resamples were drawn with replacement using seed 20260729. The polysubstance interval crossed zero; the age, sex, BPRS, motor, and non-planning intervals did not.

Supplementary Table S6. Alternative-model, influence, and duplicate-record sensitivity analyses

Model	Variable	B	95% CI	p	n
Median regression	Intercept	5.1941	2.3496 to 8.0385	<0.001	204
Median regression	Age	0.1590	0.1166 to 0.2014	<0.001	204
Median regression	Male sex	-2.6775	-3.5078 to -1.8473	<0.001	204
Median regression	Polysubstance use	1.1005	0.1385 to 2.0626	0.025	204
Median regression	BPRS total	0.2448	0.1965 to 0.2932	<0.001	204
Median regression	BIS-11 motor	0.1550	0.0564 to 0.2537	0.002	204
Median regression	BIS-11 non-planning	0.1485	0.0675 to 0.2294	<0.001	204
Fractional logit	Intercept	-1.8644	-2.0426 to -1.6861	<0.001	204
Fractional logit	Age	0.0123	0.0095 to 0.0150	<0.001	204
Fractional logit	Male sex	-0.1795	-0.2237 to -0.1353	<0.001	204
Fractional logit	Polysubstance use	0.0308	-0.0177 to 0.0794	0.213	204
Fractional logit	BPRS total	0.0163	0.0134 to 0.0192	<0.001	204
Fractional logit	BIS-11 motor	0.0147	0.0095 to 0.0199	<0.001	204
Fractional logit	BIS-11 non-planning	0.0094	0.0058 to 0.0129	<0.001	204
Exclude Cook's D > 4/n	Intercept	5.0160	2.9273 to 7.1047	<0.001	195
Exclude Cook's D > 4/n	Age	0.1865	0.1544 to 0.2186	<0.001	195
Exclude Cook's D > 4/n	Male sex	-2.9578	-3.5661 to -2.3495	<0.001	195
Exclude Cook's D > 4/n	Polysubstance use	0.8306	0.1648 to 1.4964	0.014	195
Exclude Cook's D > 4/n	BPRS total	0.2230	0.1858 to 0.2601	<0.001	195
Exclude Cook's D > 4/n	BIS-11 motor	0.2120	0.1436 to 0.2804	<0.001	195
Exclude Cook's D > 4/n	BIS-11 non-planning	0.1191	0.0696 to 0.1686	<0.001	195
Remove one record per identical pair	Intercept	3.2647	0.5370 to 5.9923	0.019	202
Remove one record per identical pair	Age	0.1861	0.1433 to 0.2290	<0.001	202
Remove one record per identical pair	Male sex	-2.6763	-3.3806 to -1.9720	<0.001	202

Model	Variable	B	95% CI	p	n
Remove one record per identical pair	Polysubstance use	0.5410	-0.2293 to 1.3114	0.169	202
Remove one record per identical pair	BPRS total	0.2429	0.1984 to 0.2874	<0.001	202
Remove one record per identical pair	BIS-11 motor	0.2222	0.1420 to 0.3025	<0.001	202
Remove one record per identical pair	BIS-11 non-planning	0.1433	0.0887 to 0.1979	<0.001	202

Note: Median-regression coefficients represent conditional median differences. Fractional-logit coefficients are on the logit scale for the aggregate score divided by 63 and use HC3 robust confidence intervals. The influence model excluded nine observations above Cook's D = 4/n; the duplicate sensitivity removed one record from each identical pair.

Supplementary Table S7. Regression diagnostics and functional-form checks

Diagnostic		Result/statistic	p
Analytic n		204	
R2 / adjusted R2		0.868 / 0.864	
Residual standard error		1.5300142969620856	
Shapiro-Wilk residuals		0.9642076328799601	<0.001
Breusch-Pagan		8.635037438030807	0.195
Ramsey RESET (squared fitted)		0.03314104959317731	0.856
Joint quadratic Wald chi-square (4 df)		11.843321677312082	0.019
Cook's D > 4/n		9	
Maximum Cook's D		0.10645039848701338	
**Quadratic sensitivity term**	**B**	**HC3 95% CI**	**p**
Age squared	-0.0055	-0.0144 to 0.0035	0.231
Male sex	-1.7126	-2.6603 to -0.7649	<0.001
Polysubstance use	0.0313	-0.9621 to 1.0246	0.951
BPRS total squared	-0.0002	-0.0062 to 0.0058	0.953
BIS-11 motor squared	0.0178	0.0062 to 0.0293	0.003
BIS-11 non-planning squared	-0.0120	-0.0246 to 0.0006	0.061

Note: The Ramsey RESET test did not indicate general misspecification, but the joint quadratic-term test was significant (chi-square = 11.843, 4 df, p = 0.019), driven mainly by the BIS-11 motor squared term. The primary linear motor coefficient is therefore an average association over the observed range.

Supplementary Table S8. Spearman correlation matrix among candidate predictors

Variable	Age	Male sex	Polysubstance	Duration ≥10	Onset <20	BPRS total	BIS motor	BIS non-planning
Age	1.00	0.05	0.28	0.85	-0.27	0.25	-0.25	-0.35
Male sex	0.05	1.00	0.41	0.14	0.24	-0.03	0.52	0.42
Polysubstance	0.28	0.41	1.00	0.19	0.25	0.70	0.67	0.56
Duration ≥10	0.85	0.14	0.19	1.00	0.00	0.28	-0.18	-0.21
Onset <20	-0.27	0.24	0.25	0.00	1.00	0.37	0.31	0.35
BPRS total	0.25	-0.03	0.70	0.28	0.37	1.00	0.50	0.39
BIS motor	-0.25	0.52	0.67	-0.18	0.31	0.50	1.00	0.83
BIS non-planning	-0.35	0.42	0.56	-0.21	0.35	0.39	0.83	1.00

Note: Binary variables were coded 1 for the named category and 0 for the reference category. The high correlations between age and duration category and between BIS-11 motor and non-planning informed the limited primary model and the additional Round 2 sensitivity analyses.

Figure S1. Residuals versus fitted values for the primary linear model

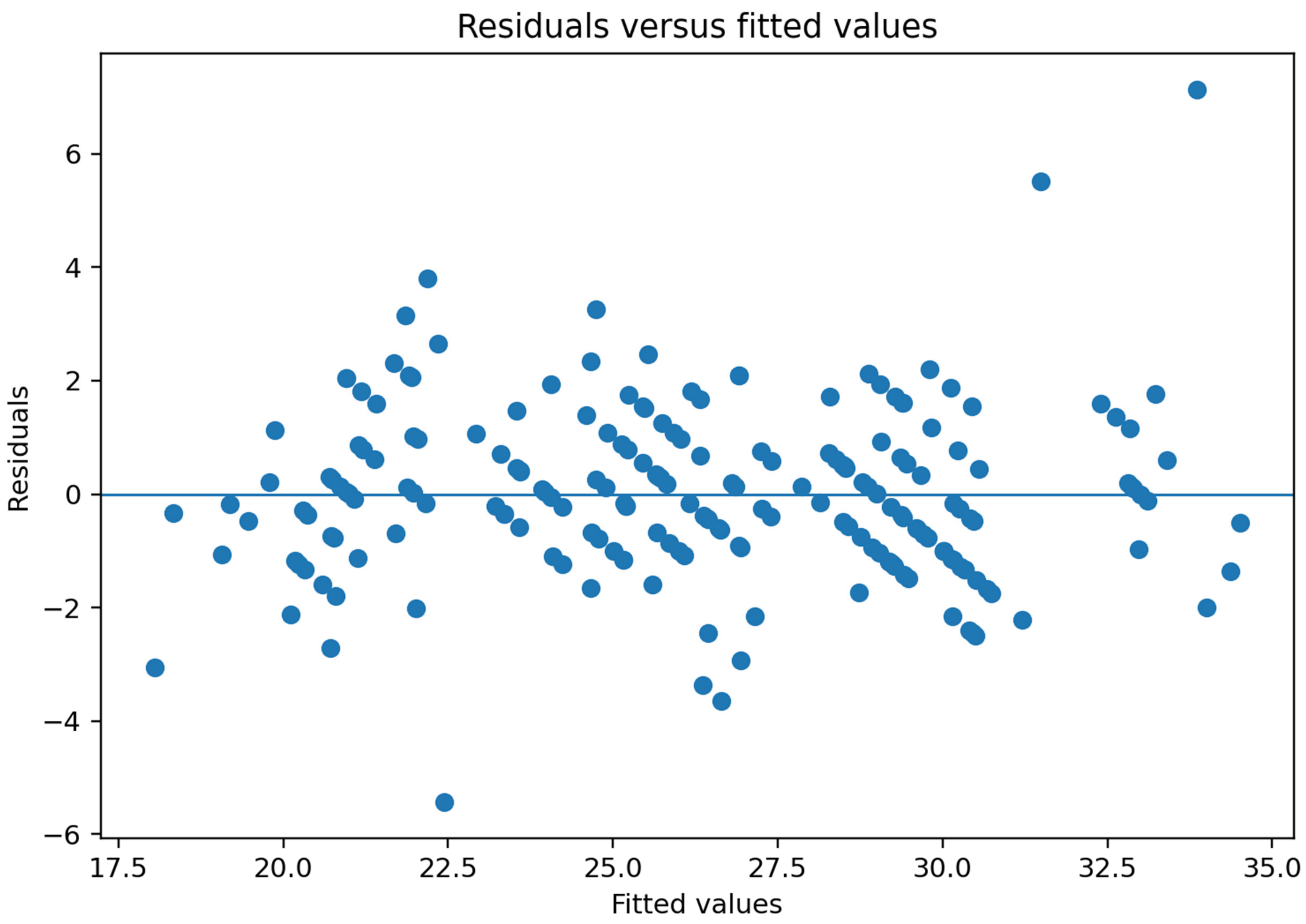

Figure S2. Normal Q-Q plot of residuals from the primary linear model

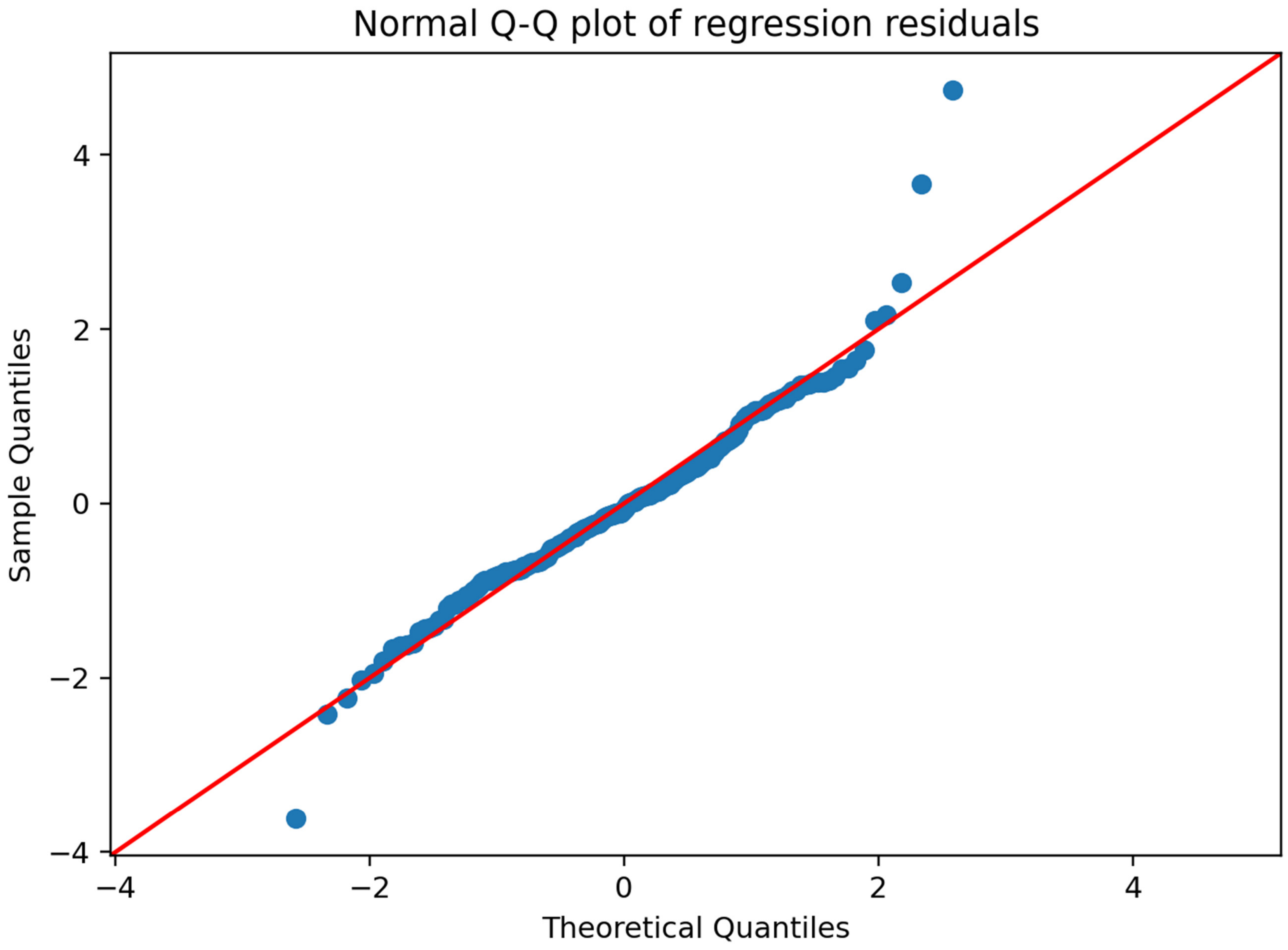

Supplement: Supplementary file 1 [file medicina-62-01504-s001.zip › medicina-4391461-supplementary.pdf]
